# Knowledge and prevalence of Human African Trypanosomiasis among residents of Kachia grazing reserve, Kachia local government area, Kaduna state, Nigeria, 2012

**DOI:** 10.11604/pamj.2016.23.89.7999

**Published:** 2016-03-15

**Authors:** Belinda Vernyuy Uba, Ahmad Aliyu, Aisha Abubakar, Sabo Ado Uba, Saheed Gidado, Aboyowa Edukugho, Ifeoma Anagbogu, John Kalejaiye, Patrick Nguku

**Affiliations:** 1 Nigeria Field Epidemiology and Laboratory Training Programm (FELTP), Abuja, Nigeria; 2 Ahmadu Bello University, Zaria (ABU), Zaria, Nigeria; 3 Federal Ministry of Health (FMOH), Abuja, Nigeria; 4 Nigeria Institute for Trypanosomiasis Research (NITR), Kaduna, Nigeria

**Keywords:** Human African Trypanosomiasis, knowledge, preventive practices, prevalence

## Abstract

**Introduction:**

Human African Trypanosomiasis (HAT) is a vector borne parasitic disease transmitted to humans by infected tse-tse flies cause morbidity including delayed child mental development. Reports of nuisance and bites from tse-tse flies by residents of Kachia grazing led to the study to determine the knowledge, practices and prevalence of HAT among residents of the grazing reserve.

**Methods:**

We conducted active case search in a cross-sectional study using multi-stage sampling with probability proportionate to size. We administered structured questionnaire on Knowledge, practices relating to HAT prevention and screened for HAT using card agglutination test for Trypanosomiasis (CATT). Knowledge of HAT was scored 0-5 and categorized good (3-5) and poor (0-2) based on score, predisposition to risk of HAT as exposure to ≥two risk factors and, a case of HAT as any respondent that tested positive on CATT. We analysed data using Epi-info and MS-excel.

**Results:**

Of the 300 respondents, mean age 39(±17years) interviewed, 56.3% were males, 12.0% had good knowledge of HAT and 76.3% were exposed to HAT risk factors. Prevention practices included clearing of overgrown bushes around houses (99%), use of insecticidal treated nets (75.7%) and protective clothing (41.0%). Males {Odds Ratio [OR] 5.0; 95% Confidence Interval (CI) 1.8 - 13.6}, age above 40 years {OR 5.0; 95% CI 1.1 - 24.4} and family history of HAT {OR 8.7; 95% CI 2.4 - 32.1} were significantly associated with HAT knowledge. None tested positive on CATT.

**Conclusion:**

Despite poor knowledge of HAT, residents practiced HAT preventive measures and zero HAT prevalence was recorded.

## Introduction

Human African Trypanosomiasis (HAT) also known as Sleeping sickness is a serious scourge to the African continent including Nigeria. It is one of the most neglected tropical diseases [1] targeted for elimination by the World Health Organization (WHO) and millions of people in 36 countries in sub-Saharan Africa are at risk of contracting the disease [2]. This re-emergence of HAT has been attributed to war, migration [3, 4] of carrier populations from active foci, environmental deterioration, increasing parasite drug resistance [3] changes of the tse-tse flies host preference, genetic variability of the parasite, the existence of asymptomatic parasite-infected individuals, and lack of surveillance plus maintenance of infection in animal reservoirs [3, 4]. In 2006, WHO noted that *Trypanosoma brucei (T.b) gambiense* caused most of the estimated 300,000-500,000 infections and Nigeria was listed amongst the countries reporting less than 100 cases [5]. The socioeconomic impact of the disease includes the loss of productivity resulting to poverty and poor socioeconomic development [6]. HAT mainly affects the poor and remote rural regions [7]. It affects children and adult and also affects men and women equally [7, 8]. People in the labour market are affected more often than others. It also affects at higher rates people who moved around a lot and those who worked in rural or domestic activities, especially those in close contact with watercourses [7]. Transmission of HAT occurs during outdoor activities like farming, hunting, fishing or washing of clothes [8]. HAT is a debilitating disease which if left untreated can result in high fatality rates [1, 5]. Human infections reduce labour resources, while the animal disease limits availability of meat and milk and deprives African farmers of draught animal power, substantially minimising crop production. Children affected by the disease suffer considerable delay in their mental development which impacts negatively on their school performance and professional advancement [9]. The prevalence of HAT in the reserve is unknown despite persistent reports of nuisance and bites by tse-tse flies from the residents of the grazing reserve who are mostly at risk as they live with their livestock and work with them near rivers and streams during both wet and dry season in areas infested by tse-tse flies. Due to the dearth of literature on the prevalence of HAT in the Kachia grazing reserve, the lack of a systematic active surveillance system for HAT in some parts of the country as documented by WHO [10] and the need to determine the HAT situation in this locality this study was conducted to determine the knowledge of HAT and provide baseline information on the prevalence of HAT in the grazing reserve to informed decision making in the implementation of prevention and control measures.

### Specific Objectives

To determine the knowledge of HAT among residents of Kachia grazing reserve; to determine the exposure to risk factors for HAT infection among residents of Kachia grazing reserve; to determine the prevalence of HAT among residents of Kachia grazing reserve; to determine practices relating to HAT prevention among residents of Kachia grazing reserve.

## Methods

A cross sectional descriptive study was conducted between January to June 2012 among residents of Kachia grazing reserve found in Kachia Local Government Area (LGA) of Kaduna State about 90 kilometers from Kaduna town in North Western Nigeria with an estimated population of about 24,500 [11]. Residents who had resided in the reserve had for at least 1year and consented to participate in the study were included. This duration of stay would have predisposed them to the tse-tse flies as they move about their normal activities. Sample size calculation for a descriptve study [12] was used to calculate the sample size using the 2006 prevalence of HAT in endemic focus of Delta state, Nigeria as documented by the Federal Ministry of Health [13] at 95% confidence interval and 5% level of significance (α). A multistage sampling technique was used with probability proportionate allocation of sample size. In stage one Settlements were randomly selected proportionately from each of the six blocks of the grazing reserve. In the second stage, households were systematically sampled for screening. The first household was randomly selected and the kth household was then systematically sampled until the required number of sample was obtained. The sampling interval (k) was obtained by dividing the total number of households in the selected settlements (520) by the total number of households to be sampled (150). This gave the sampling interval of 4. In the third stage, eligible members of selected households were randomly selected by balloting. interviewer administered questionnaire was used to obtain socio-demographic information of the respondents, knowledge of HAT and exposure to known risk factors for HAT transmission and practices among respondents relating to HAT prevention. The Card Agglutination Test for Trypanosomiasis (CATT) kit was used to screen respondents for circulating HAT antibodies that are produced following infection by *T.b. gambiense* using whole blood. Questionnaire were interviewer administered following which consented interviewed respondents were finger pricked and blood collected for HAT screening using CATT test kits. Data was collected on socio demographic characteristics of respondents, knowledge of HAT (what HAT is, the causative agent, mode of transmission, signs and symptoms, prevention) and practices relating to HAT prevention among respondents. Knowledge of HAT by the respondents was graded on a scale into five points. A point was scored for each correct response to a question and zero for wrong response with a maximum score of five and a minimum score of zero. Total score of 0 to 2 was graded as poor knowledge while score of 3 to 5 was graded as good knowledge.

**Exposure status**: residents exposed to two or more of risk factors for HAT were regarded as being exposed to risk of HAT transmission.

**HAT prevalence**: the HAT prevalence among the respondents was determined based on CATT positive test result. A case of HAT was defined as any respondent that tested positive on CATT. Data collected was entered, cleaned and analysed using Microsoft excel 2007 and Epi-Info software version 3.5.3. Univariate analysis was performed for frequencies and proportions and bivariate analysis for associations related to knowledge of the HAT and preventive practices. Ethical clearance was obtained from the Ahmadu Bello University, Ethical Committee and the Kaduna State Ministry of Health. Permission was also obtained from the district head of Ladduga. Informed consent was obtained from respondents and from parents of minors in addition to ascent before the study and data confidentiality was maintained by keeping the questionnaire that had the respondents name with the researcher alone, not releasing any information regarding respondents and only entering questionnaire numbers in the data base.

### Limitations

One of the limitations to this study was the sensitivity of the CATT reagent of 92% - 98% [14] as documented by Chappuis in the Field diagnosis of HAT.

## Results

The mean age of respondents was 39 ± 17years and 62.7% of respondents were below forty years and majority (56.3%) were males. Most respondents (86.3%) were married; only 14.7% secondary and post-secondary education and majority 44.3% practiced mainly animal husbandry as a means of livelihood. Most (93.3%) of respondents were Fulanis and the mean duration of residence in the grazing reserve was 22 (± 8years), range of 1-33years (Table 1).

**Table 1:** Socio demographic characteristic of respondents in Kachia grazing reserve, Kachia LGA, Kaduna State, Nigeria, 2012 [n = 300]

Variable	Frequency	Percent [%]
Mean age 39±17years		
**Age Group[Years]**		
<40	188	62.7
≥40	112	37.3
**Sex**		
Female	131	43.7
Male	169	56.3
**Marital Status**		
Single	35	11.7
Married	259	86.3
Widowed	5	1.7
Divorced	1	0.3
**Educational Qualification**		
No Formal Education	199	66.3
Primary	57	19.0
Secondary	38	12.7
Post-Secondary	6	2.0
**Occupation**		
Farming	14	4.7
Husbandry	133	44.3
Trading	87	29.0
Artisan	18	6.0
Civil Servant	6	2.0
Unemployed	42	14.0
**Tribe**		
Fulani	280	93.3
Hausa	20	6.7

### Knowledge of HAT

About 56(18.7%) had Knowledge of what HAT is and its signs and symptoms, 17.3% knew the causative agent and 12.0% knew mode of transmission. On knowledge grading, only 36(12%) respondents had good knowledge of HAT.

### Exposure to risk factors for HAT transmission

Exposure to predisposing factors for HAT was quite prevalent among respondents. Two hundred and twenty nine (76.3%) respondents were exposed to at least two or more risk factors. The commonest exposure were washing clothes in the stream (72.7%), bathing in the stream (71.0%), fetching water from the stream (57.7%) and fetching of firewood from the bush (55.0%) amongst others.

### Prevalence of HAT

Of the 300 respondents that were examined and screened for HAT, none had palpable cervical lymph nodes enlargement and none tested positive on CATT; hence HAT prevalence was zero.

### Practices relating to HAT prevention

These included clearing of overgrown bushes around houses (99%) to use of insecticidal treated nets (ITNs) (76%) and use of protective clothing when visiting the bush (41%).

### Determinants of knowledge of HAT (Table 2)

Male respondents (OR4.5, 95% CI 1.7-12.5, p 0.0009), educated (post-secondary) (OR 5.0, 95%CI 2.3-10.7, P 0.00003), ≥40years of age (OR 2.7, 95%CI 1.3-5.4, p 0.009), resident in the grazing reserve for ≥2 years (OR 2.8, 95%CI 1.3-6.2, p 0.01), had good knowledge of AT (OR 2.5, 95%CI 1.2-5.2, p 0.02), past family history of HAT (OR 6.4, 95%CI 2.1-19.7, p 0.001), had significant associations with knowledge of HAT and prevention practices. Occupation such as farming and animal husbandry (OR 2.0 95%CI 1.00-4.0, p 0.08) were not significantly associated with knowledge of HAT. Same factors significantly associated with HAT prevention practices. An unconditional logistic regression model was used to fit in variables in a stepwise manner and the following factors; males (AOR 5, 95%CI 1.8-13.6, p 0.002), ≥40years of age (AOR 5, 95%CI 1.1-24.4, p 0.05), family history of HAT (AOR 8.7, 95%CI 2.4-32.1, p 0.001) except >2yrs duration of stay in the reserve (AOR 2.2, 95%CI 1.0-5.1, p 0.06) were found to be determinants of knowledge of HAT among residents of the reserve (Table 3). Same model was used to identify determinants HAT prevention practices among residents. Males (AOR 4.2, 95%CI 1.8-10.0, p 0.001), family history of HAT (AOR 6.2, 95%CI 1.9- 23.7, p 0.003), >2yrs of resident in the reserve (AOR 2.9, 95%CI 1.4-6.2, p 0.006), and educated respondents (AOR 4.4, 95%CI 2.2- 9.6, p 0.0002) were significant determinants of practices relating to HAT prevention.

**Table 2:** Association between Socio- demographic characteristics and knowledge of HAT among respondents in Kachia grazing reserve, Kachia LGA, Kaduna State, Nigeria, 2012 [n = 300]

Variable	Knowledge		Odds Ratio [95% CI]	p-value
	Good [%]	Poor [%]	
**Sex**				
Male	30[83.3]	139[52.7]	4.5[1.7 – 12.5]	0.0009
Female	6[16.7]	125[47.3]		
**Educational Qualification**				
Educated [Secondary & post]	14[38.9]	30[11.4]	5.0[2.3-10.7]	0.00003
Not educated [none & primary]	22[61.1]	234[88.6]		
**Age group**				
≥40yrs	21[58.3]	91[34.5]	2.66[1.31-5.41]	0.009
<40yrs	15[41.7]	173[65.5]		
**Occupation**				
Farming/Husbandry	23[63.9]	124[47.0]	2.0[1.00-4.11]	0.08
Others	13[36.1]	140[53.0]		
**Duration of stay in the area**				
>2yrs	27[75.0]	136[51.5]	2.82[1.28-6.23]	0.01
≤2yrs	9[25.0]	128[48.5]		
**Knowledge of AT**				
Good	24[66.7]	117[44.3]	2.51[1.21-5.24]	0.02
Poor	12[33.3]	147[55.7]		
**Family History of HAT**				
Yes	6[16.7]	8[3.0]	6.4[2.08-19.70]	0.001
No	30[83.3]	256[97.0]		

**Table 3:** Unconditional logistic regression factors associated with good knowledge of HAT among respondents in Kachia grazing reserve, Kachia LGA, Kaduna State, Nigeria, 2012

Variable	Adjusted OR	95% CI	P- Value
Male	5.0	1.8, 13.6	0.002
Age ≥ 40years	5.0	1.0, 24.4	0.05
Family history of HAT	8.7	2.4, 32.1	0.001
≥2years of Residence	2.2	1.0, 5.1	0.06

## Discussion

This study looked into the knowledge and prevalence of HAT among residents of Kachia grazing reserve and practices among residents relating HAT prevention. Majority of the respondents of the Kachia grazing reserve had poor knowledge of HAT and were mostly exposed to risk factors for the disease. The prevalence of HAT among residents of the Kachia Grazing reserve of Kaduna State was zero. Respondents frequently engaged in common practices related to HAT prevention. The socio demographic characteristics of respondents showed that most of the respondents were Fulanis. This is typical of a grazing reserve meant for animal husbandry in Nigeria. Male predominance is typical of most studies in sub Saharan Africa with majority of respondents in the active age group 20-49years. Participation of females was reasonably high, probably due to the socialization of an average Fulani woman as they do interact freely with the public while carrying out their trade. The educational qualification of respondents showed that most of them do not have formal education; only 14.7% had secondary and post-secondary education. This is far below the national adult literacy level [15]. A review of marital status of respondents showed that majority are married. This is a common practice amongst the fulanis where marriage is a rule, and they do so very early in life. The occupational status of respondents showed that most of them were cattle rearers [16] which is expected of a grazing reserve. Majority of the female respondents were into trading. This is the normal trade of Fulani women in cows’ milk called “Fura da Nunu” in local language. Most residents have resided in the grazing reserve for two or more years. This is because grazing reserves in Nigeria are areas demarcated by government for grazing where Fulanis can settle permanently without being disturbed by farmers [17]. A review of general knowledge of HAT showed that respondents’ knowledge of HAT was poor. This is also similar to studies conducted on HAT among nomadic Fulanis in Northern Nigeria in which knowledge of the disease was poor [16]. Also similar to a related study in the DR Congo in which knowledge was poor among the respondents [16, 18]. This shows that even though they are exposed, they are not aware of risk associated with the exposure. An in-depth review of knowledge of HAT commonly known as ‘Ciwon bachi’ in local language showed that knowledge of the causative agent, the mode of transmission and symptoms of HAT was quite poor. This is however, contrary to a similar study conducted in Zambia in which most of the respondents who had lived in the area for at least one month knew that tsetse fly bites transmitted trypanosomiasis [19] and in a related study in the Serengeti National Park Tanzania, most residents knew sleeping sickness, the right place to seek healthcare and that sleeping sickness infections were acquired in the bush and forest through a tsetse bite [20].

In this study, the lack of good knowledge could be due to non endemicity of the disease in the reserve since no institutionalized measures had been put in place to enlighten the residents about the disease and its prevention and control measures. Despites the poor knowledge of HAT, a greater proportion of respondents of the grazing reserve had satisfactory knowledge of AT as lots of work on animal trypanosomiasis has been going on in the grazing reserve [21, 22]. The association of gender, age of respondents, duration of stay at the reserve and family history of HAT with knowledge of HAT and implementation of preventive practices is quite similar to the Kinshasa study [18] in which males, adults and residence in an endemic area for more than 3months were associated with knowledge of HAT. Exposure to known risk factors that could predispose residents to HAT was reviewed among respondents. Risk factors explored were engagement in activities like farming, hunting, fishing, fire wood fetching in an area infested by tse-tse flies that have been documented [2, 8, 23, 24]. Water contact activities such as washing clothes/bathing in the stream or fetching water believed to carry a high risk for HAT infection due to the proximity to the vector habitat were also considered [25]. A detailed review of exposure to predisposing factors for HAT showed that majority of respondents were exposed to at least two or more factors. Most respondents exposed were aged 20- 49 years who are the active population in the labour market and move around a lot in the reserve as they graze their animals, farm and perform other rural and domestic activities similar to the study in Kinshasa [7] Mostly male respondents were exposed than females. This is likely due to the engagement of males in the Northern part of the country in most outdoor activities while the females stay indoor though studies have shown that there is no gender difference in exposure [7]. Respondents frequently engaged themselves in activities like washing in the stream, bathing in the stream, firewood fetching and hunting which exposed them to contact and possibly bites by tse-tse flies. Review of HAT prevalence in the grazing reserve following CATT screening showed zero prevalence of HAT among the respondents even though there had been reported episodes of contact and bites by tse-tse flies. This is similar to a prevalence study conducted in Northern Nigeria among migrant fulanis around Lake Kainji area on HAT in which no case was recorded even though it was noted that the transhumance pastoral mobility of these migrant fulanis could be an important factor in any outbreak of human trypanosomiasis around Kainji Lake area in future [16]. Considering non infectivity of the vector as a possible explanation, only tse-tse infected with *T. brucei gambiense* causative agent for the gambiense form of HAT can transmit infections to humans following bites [9]. This is supported by the report of a survey on sero prevalence of AT in the grazing reserve in 2009 in which vector parasitology showed that the vector (tse-tse) were not infected with T.b. gambiense rather, they harboured the infective agents for AT *(T. vivax, T. congolense and T. b. brucei)* that had led infection of animals with high AT prevalence recorded [22]. The presence of domestic animals in the reserve and the role played by these domestic animal reservoirs in parasite transmission of HAT to humans has been documented to be unclear as to whether they increase the transmission to human or on the contrary act as bait thus providing some degree of protection [26] to human and needs to be further evaluated.

Also to be considered is the genetic susceptibility of an individual to infection which is controlled by genetic factors from the host or by the parasite's virulence [27]. The existence of an individual susceptibility to HAT known as trypanotolerance [28, 29] has been suspected and documented. Trypanotolerance which has been well described in animal trypanosomiasis [26, 30] appears to also occur in humans as some individuals are able to somehow control *T. b. Gambiense* infections [26]. This phenomenon probably may have occurred among residents of the grazing reserve who at one time might have been infected, got to control the infection and became self-cured hence the negative test results. This could possibly explain the zero prevalence detected in the reserve. This is also supported by the study conducted by Bucheton et al on the long-term follow-up of 11 HAT case patients that refused treatment in Côte d'Ivoire for fifteen years which led to the identification of these cases who were initially diagnosed in first stage by microscopy, yet on follow-up examination had no detectable clinical symptoms and no detectable parasitaemia by microscopy. Subsequent long-term follow-up showed a drop in antibody titres to sero negative levels in these cases, indicating that they had self-cured [26]. Also, studies on human genetic factors influencing infection by *T. b. Gambiense* looked at polymorphisms in cytokine genes and the development of HAT. These studies showed, on one hand, a significant association with polymorphisms located in the IL-6 and IL-10 genes and a decreased risk of developing HAT and, on the other hand, a significant association between polymorphisms located in the IL-1a and TNF-a genes and an increased risk of developing the disease [26, 31, 32]. These factors needs to be examined further in this context of zero prevalence obtained in the grazing reserve. Findings from the study revealed that practices relating to HAT prevention are common among almost all of the residents especially the usage if ITNs. This could also explain the non prevalence of HAT amongst them as they often protect themselves from tse-tse bites. A review of common practices to avoid contact or being beaten by tse-tse flies among respondents showed that clearing of overgrown bushes around their houses was a common practice in almost all of the respondents. This was aimed at preventing infestation of these areas by tse-tse flies. Also majority of respondents used insecticidal treated nets in their homes and protective clothing when visiting the bush and tse-tse infested areas for hunting or grazing. Even though respondents’ knowledge of the disease cause by the vector was poor, they still put in adequate preventive measures to protect themselves from this vector that transmit disease. This is similar to the finding reported in the study conducted on HAT in communities around the Serengeti Park in Tanzania [20]. These are preventive measures that are advocated for HAT disease prevention and control. A review of the health seeking behaviour among respondents showed that some respondents knew that the right place to seek healthcare was the hospital if ill as had been documented in similar studies in the Serengeti Park in Tanzania [20]. Notably was the finding that most of respondents did not know where to seek medical help if ill. This finding is contrary to studies in Kinshasa DR Congo whereby a greater proportion of respondents would resort to churches for help because of their belief in the supernatural cause of HAT [18]. In the case of health seeking behaviour for their sick animals, almost all respondents would seek help from a veterinarian for their sick animals and only a few reported doing nothing to their sick animals. These are pastoralists who place their animals at very high esteem.

## Conclusion

In this study we can conclude that Knowledge of HAT among residents of Kachia grazing reserve is poor, there is no ongoing transmission of HAT in the reserve and most of the residents are exposed to risk factors for disease transmission in the reserve. This calls for concerted efforts by government at all levels including the relevant line ministries to put in place vector control measures to avert future occurrence of disease outbreak in an event there is introduction of HAT in the grazing reserve.

### What is known about this topic

- The knowledge of HAT is poor among residents of the grazing reserve.
- There is no ongoing transmission of HAT in the grazing reserve.
- The residents of the grazing reserve are highly exposed to tse-tse flies putting them at risk of disease transmission and future outbreaks if there is introduction of the human infective form of trypanosome.

### What this study adds

- The estimated prevalence of HAT in the grazing reserve is known and serves as a baseline for future studies.
- It has further consolidated the fact that human exposure to the vector carrying animal infective form of trypanosome (as documented in other studies) does not invariably cause HAT.
- It has provided information for policy makers to redirect their limited resources towards the control of Animal trypanosomiasis in the grazing reserve.
